# Hyperbaric Oxygen Attenuates Cerebral Ischemia–Reperfusion Injury Through ROS-Dependent Remodeling of Microglial Mitochondrial Dynamics

**DOI:** 10.3390/ijms27146334

**Published:** 2026-07-16

**Authors:** Haotian Wei, Xingyue Du, Shushu Xu, Qiuli Bo, Yanan Guo, Lihua Xu, Zhenglin Jiang, Xia Li, Yuan Yuan

**Affiliations:** Institute of Special Environmental Medicine, Co-Innovation Center of Neuroregeneration, Nantong University, Nantong 226019, China; 2325310025@stmail.ntu.edu.cn (H.W.); dxy@stmail.ntu.edu.cn (X.D.); 2325310011@stmail.ntu.edu.cn (S.X.); Boqiuli@stmail.ntu.edu.cn (Q.B.); 2123310002@stmail.ntu.edu.cn (Y.G.); xulihua0527@ntu.edu.cn (L.X.); jiangzl@ntu.edu.cn (Z.J.)

**Keywords:** hyperbaric oxygen, cerebral ischemia–reperfusion injury, microglia, reactive oxygen species, mitochondrial dynamics, neuroinflammation

## Abstract

Hyperbaric oxygen (HBO) shows neuroprotective potential in cerebral ischemia–reperfusion (CIR) injury, but its variable efficacy suggests that the underlying cellular mechanisms remain incompletely defined. We previously showed that HBO suppresses microglial NLRP3 inflammasome activation after CIR injury in a reactive oxygen species (ROS)-dependent manner; yet, how ROS couples to this effect remains unclear. Since mitochondria regulate ROS and inflammasome signaling, we investigated whether HBO modulates microglial mitochondrial dynamics in CIR injury. In adult male ICR mice (n = 71, 8–12 weeks) subjected to 60 min middle cerebral artery occlusion followed by 24 h reperfusion, HBO improved neurological function, reduced infarct area, and decreased ASC-positive microglia/macrophages. In lipopolysaccharide/nigericin-stimulated primary microglia, HBO suppressed IL-1β release, reduced mitochondrial fragmentation, preserved mitochondrial membrane potential, maintained mitofusin 2 (MFN2) protein level, and reduced DRP1 Ser616 phosphorylation without altering total DRP1 or FIS1 expression. MitoTEMPOL abolished HBO-mediated protection against mitochondrial fragmentation, MFN2 reduction, and DRP1 Ser616 phosphorylation in vitro. Edaravone, when combined with HBO, attenuated HBO-mediated neuroprotection and counteracted HBO-induced regulation of MFN2 and DRP1 Ser616 phosphorylation in vivo. These findings support ROS-dependent remodeling of microglial mitochondrial dynamics as a mechanism contributing to HBO-mediated suppression of inflammasome-associated inflammation after CIR injury.

## 1. Introduction

Stroke is a major acute cerebrovascular disease and remains one of the leading causes of death and long-term disability worldwide. Ischemic stroke accounts for the majority of stroke cases. Although timely reperfusion is essential for salvaging ischemic tissue, restoration of blood flow can paradoxically trigger cerebral ischemia–reperfusion (CIR) injury, which contributes to secondary brain damage and limits functional recovery [1]. Despite substantial advances in reperfusion strategies, CIR injury remains a major unmet challenge during the acute phase of ischemic stroke, highlighting the need for effective adjunctive therapies that can reduce reperfusion-associated injury and improve functional recovery.

Hyperbaric oxygen (HBO) therapy, defined as the administration of pure oxygen at pressures exceeding one atmosphere absolute, has demonstrated neuroprotective effects across a wide range of experimental models of neurological injury [2,3,4]. Increasing evidence indicates that HBO ameliorates CIR injury by mitigating oxidative stress, reducing inflammation, and enhancing neural repair [5,6,7]. However, clinical outcomes have been variable, and the precise cellular and molecular mechanisms underlying HBO-mediated neuroprotection remain incompletely understood. Neuroinflammatory responses, particularly those mediated by microglia, may represent important determinants of HBO responsiveness and key mechanistic targets of HBO therapy.

Microglia, the principal immune cells in the central nervous system, play a pivotal role in post-ischemic neuroinflammation. Following CIR, excessive microglial activation promotes secondary injury by releasing proinflammatory mediators that compromise neuronal survival and blood–brain barrier integrity [8]. Among the signaling pathways involved, the NOD-like receptor protein 3 (NLRP3) inflammasome has emerged as a critical inflammatory hub that amplifies neuroinflammation and worsens outcomes following CIR [9]. In our previous work, we demonstrated that HBO treatment improved neurological function and reduced hemorrhagic transformation in a hyperglycemia-associated transient middle cerebral artery occlusion (tMCAO) model. These effects were at least partly mediated by suppression of microglial NLRP3 inflammasome activation and the downstream inflammatory cascade [10]. Notably, this anti-inflammatory effect was dependent on reactive oxygen species (ROS), as antioxidants such as edaravone (Eda) and N-acetyl-L-cysteine (NAC) reversed HBO-induced inhibition of microglial activation and attenuated its neuroprotective effects in vivo [10]. However, whether this ROS-related anti-inflammatory mechanism also operates in conventional CIR injury and how ROS signaling is translated into microglial inflammasome regulation remain unclear.

Mitochondria provide a biologically plausible link between ROS-related signaling and inflammasome regulation. As major sources and sensors of intracellular ROS, mitochondria not only contribute to oxidative stress but also serve as signaling organelles that coordinate cellular stress responses, bioenergetic adaptation, and inflammatory activation [11,12]. In microglia, mitochondrial dysfunction can promote NLRP3 inflammasome activation through excessive mitochondrial ROS production, loss of mitochondrial membrane potential, impaired metabolic homeostasis, and release of mitochondria-associated danger signals [11,12]. Mitochondrial dynamics further shape this regulatory interface, as the mitochondrial network is continuously remodeled by the balance between fission and fusion, governed by fission mediators such as DRP1 and FIS1 and fusion proteins such as MFN1, MFN2, and OPA1 [13].

Excessive fission or impaired fusion can lead to mitochondrial fragmentation, membrane potential collapse, increased mitochondrial ROS accumulation, and enhanced inflammasome-associated inflammatory activation [13,14]. Conversely, preservation of mitochondrial network integrity may limit mitochondrial stress and suppress inflammatory signaling. Because mitochondrial fission and fusion are highly sensitive to redox changes [15,16], ROS may serve not only as damaging molecules but also as upstream signals that reshape mitochondrial dynamics. Dysregulation of mitochondrial dynamics has been reported in ischemic and inflammatory conditions [13,17], yet whether HBO modulates mitochondrial dynamics regulators in microglia after CIR injury remains unknown.

In the present study, we tested the hypothesis that HBO attenuates CIR injury by remodeling microglial mitochondrial morphology and dynamics through ROS-related signaling, thereby limiting inflammasome-associated inflammatory responses. Elucidating this mechanism may deepen our understanding of HBO-mediated neuroprotection and provide a rational basis for optimizing HBO-based therapeutic strategies for acute ischemic stroke.

## 2. Results

### 2.1. HBO Treatment Attenuates Cerebral Ischemia–Reperfusion Injury and Inflammasome Activation in Microglia/Macrophages

**Figure 1:**
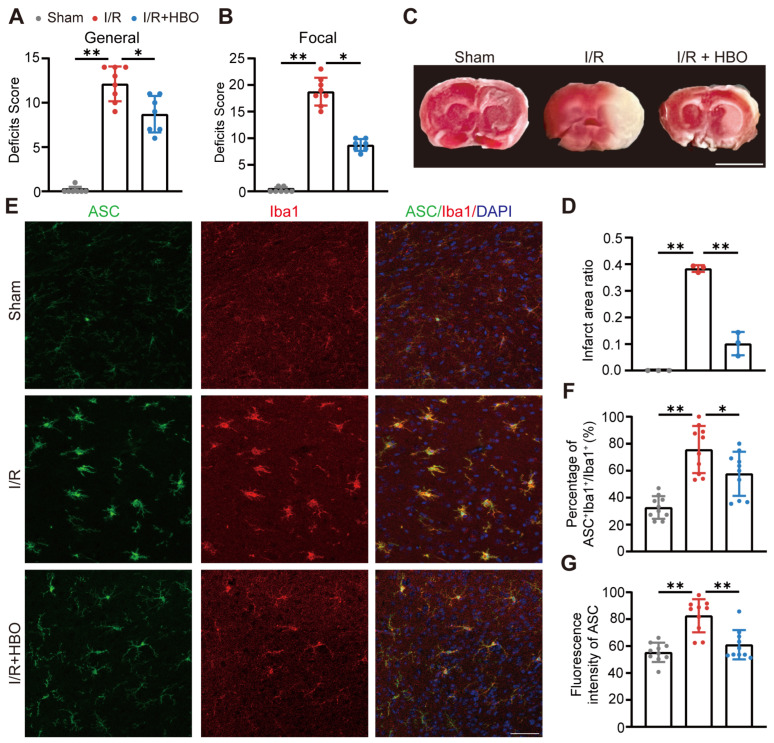
HBO treatment attenuates cerebral ischemia–reperfusion injury and inflammasome activation in microglia/macrophages: (**A**,**B**) General (**A**) and focal (**B**) neurological deficit scores of mice in each group (n = 7–8). (**C**) Representative TTC-stained brain sections from each group. Scale bar = 0.5 cm. (**D**) Quantification of infarct area in each group (n = 3). As shown in (**A**–**D**), HBO treatment mitigated cerebral ischemia–reperfusion injury in the tMCAO model compared with the I/R group. (**E**) Representative double-immunofluorescence images of the peri-infarct striatal region after tMCAO showing Iba1-positive microglia/macrophages (green) and ASC (red); nuclei were counterstained with DAPI (blue). The colocalization of Iba1 and ASC signals is represented in yellow. Scale bar = 50 μm. (**F**,**G**) Quantitative analyses of the percentage of ASC-positive Iba1-positive cells among total Iba1-positive microglia/macrophages (**F**) and the mean fluorescence intensity of ASC in Iba1-positive cells (**G**) in each group. Each data point represents one field of view (FOV). Data were obtained from 3 mice, with 3–4 FOVs analyzed per mouse. HBO inhibited inflammasome activation in microglia/macrophages. The Kruskal–Wallis test followed by Dunn’s multiple comparisons test with Benjamini, Krieger, and Yekutieli FDR correction was used for (**A**,**B**,**G**), and one-way ANOVA followed by Tukey’s multiple comparisons test was used for (**D**,**F**). Data are presented as mean ± SD. * *p* < 0.05, ** *p* < 0.01. I/R: ischemia/reperfusion; HBO: hyperbaric oxygen; ASC: apoptosis-associated speck-like protein containing a CARD; Iba1: ionized calcium-binding adaptor molecule 1; DAPI: 4′,6-diamidino-2-phenylindole.

To confirm the neuroprotective effect of HBO treatment, we evaluated neurological deficits in the tMCAO model. Consistent with our previous findings in a hyperglycemia-enhanced hemorrhagic transformation model [10], HBO treatment alleviated behavioral abnormalities, as reflected by reduced general and focal deficit scores in the ischemia/reperfusion (I/R) + HBO group compared with the I/R group (Figure 1A,B; *p* < 0.05). In addition, HBO treatment decreased infarct area, as assessed by TTC staining (Figure 1C,D; *p* < 0.01), further confirming that HBO attenuates cerebral ischemia–reperfusion (CIR) injury.

To determine whether HBO modulates microglial/macrophage inflammasome activation in vivo, we examined ASC, an essential adaptor protein of the NLRP3 inflammasome complex, in Iba1-positive microglia/macrophages. Immunofluorescence staining showed that HBO treatment reduced the proportion of ASC-positive Iba1-positive cells among total Iba1-positive microglia/macrophages (Figure 1E,F; *p* < 0.05), as well as ASC fluorescence intensity (Figure 1G; *p* < 0.01), in the I/R + HBO group compared with the I/R group. These results indicate that HBO suppresses inflammasome activation in microglia/macrophages after tMCAO, consistent with our previous findings [10].

### 2.2. HBO Suppresses LPS/Nigericin-Induced Mitochondrial Fragmentation and Preserves Mitochondrial Membrane Potential in Microglia

**Figure 2:**
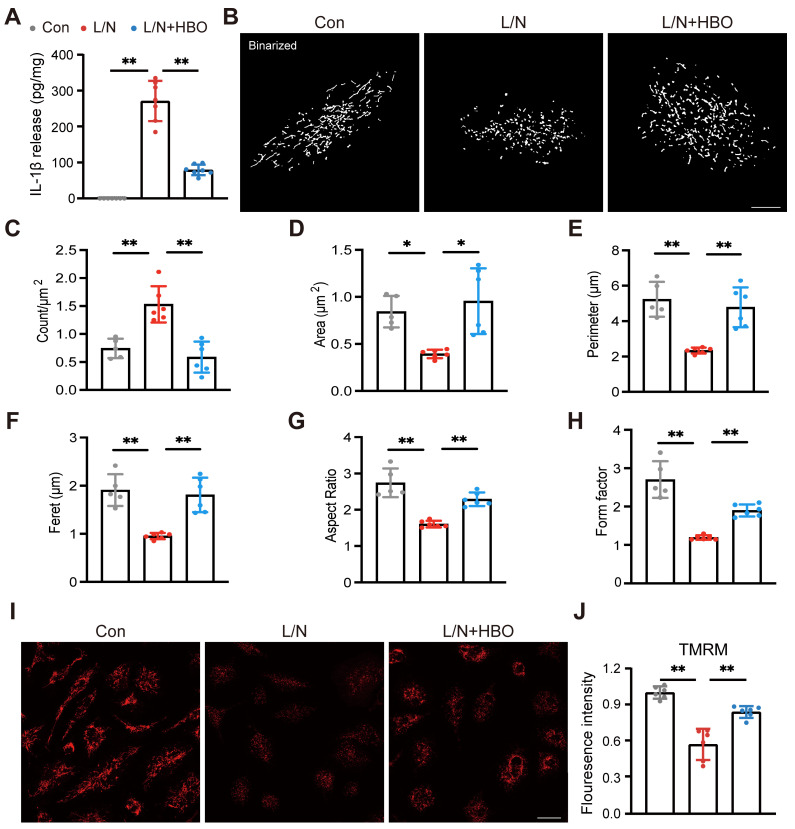
HBO suppresses LPS/nigericin-induced mitochondrial fragmentation and preserves mitochondrial membrane potential in microglia: (**A**) Levels of IL-1β released by primary microglia in different treatment groups (n = 7). HBO inhibited IL-1β release in L/N-stimulated microglia compared with the L/N group. (**B**) Representative binarized images of TMRM-labeled mitochondria showing mitochondrial morphology in microglia under different treatment conditions. Scale bar = 10 μm. (**C**–**H**) Quantitative analyses of mitochondrial parameters, including mitochondrial number per μm^2^ (**C**), mean area (**D**), mean perimeter (**E**), mean Feret diameter (**F**), aspect ratio (**G**), and form factor (**H**) (n = 5–6). HBO attenuated L/N-induced mitochondrial fragmentation in microglia. (**I**) Representative images of TMRM-labeled mitochondria (red). Scale bar = 20 μm. (**J**) Quantitative analysis of TMRM fluorescence intensity in microglia from each group (n = 6). HBO prevented the decrease in mitochondrial membrane potential in L/N-stimulated microglia compared with the L/N group. Welch’s ANOVA followed by Dunnett’s T3 multiple comparisons test was used for (**A**,**D**,**F**), and one-way ANOVA followed by Tukey’s multiple comparisons test was used for (**C**,**E**,**G**,**H**,**J**). Data are presented as mean ± SD. * *p* < 0.05, ** *p* < 0.01. Con: control; L/N: lipopolysaccharide/nigericin; HBO: hyperbaric oxygen; IL-1β: interleukin-1β; TMRM: tetramethylrhodamine methyl ester.

To further elucidate the cellular mechanisms underlying the anti-inflammatory effect of HBO, we investigated whether HBO directly modulates microglial activation and mitochondrial dynamics in vitro. Consistent with our previous report [10], HBO treatment inhibited IL-1β release, a marker of NLRP3 inflammasome activation, in primary microglia stimulated with LPS and nigericin (L/N) (Figure 2A; *p* < 0.01), confirming a direct inhibitory effect on microglial inflammatory activation.

Given the established role of mitochondrial fragmentation in NLRP3 inflammasome activation [14], we next examined mitochondrial morphology using TMRM labeling. In the L/N stimulation model, microglia exhibited pronounced mitochondrial abnormalities, including increased mitochondrial number and fragmentation. HBO treatment markedly reduced the L/N-induced increase in mitochondrial number (Figure 2B,C; *p* < 0.01) and preserved mitochondrial network integrity, as reflected by significant improvements in multiple morphological parameters, including mean area, perimeter, Feret diameter, aspect ratio, and form factor (Figure 2D–H; all *p* < 0.05 or *p* < 0.01). In addition, HBO treatment preserved mitochondrial membrane potential, as indicated by sustained TMRM fluorescence intensity (Figure 2I,J; *p* < 0.01). Together, these results indicate that HBO suppresses L/N-induced mitochondrial fragmentation and preserves mitochondrial function in microglia.

### 2.3. HBO Prevents MFN2 Reduction and Suppresses DRP1 Phosphorylation at Ser616 in LPS/Nigericin-Stimulated Microglia

To identify the molecular targets through which HBO regulates mitochondrial dynamics, we examined the expression of key mitochondrial fusion-related proteins, including MFN1, MFN2, and the long isoform of OPA1 (L-OPA1), the functionally active form required for maintaining inner mitochondrial membrane fusion [18,19]. We also examined mitochondrial fission-related proteins, including DRP1 and FIS1 [13,20], by Western blot.

**Figure 3:**
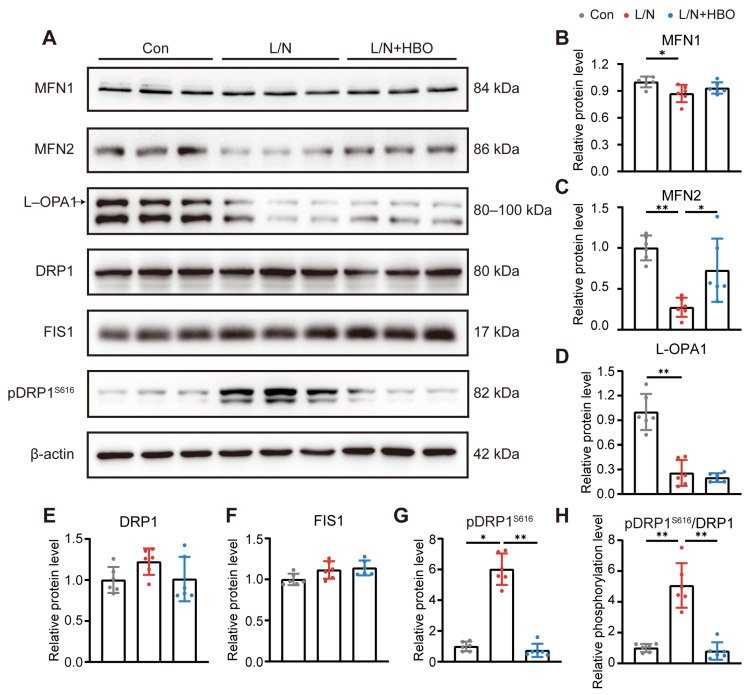
HBO prevents MFN2 reduction and suppresses DRP1 phosphorylation at Ser616 in LPS/nigericin-stimulated microglia: (**A**) Representative immunoblot images showing the protein levels of MFN1, MFN2, OPA1, DRP1, FIS1, and DRP1 phosphorylation at Ser616 (pDRP1^S616^). OPA1 appears as two major isoforms: long OPA1 (L-OPA1, upper band, arrow) and short OPA1 (S-OPA1, lower band). Quantitative analysis in this study was performed specifically on L-OPA1, the functionally active isoform involved in mitochondrial fusion. β-actin served as the loading control. (**B**–**H**) Quantitative analyses of MFN1 (**B**), MFN2 (**C**), L-OPA1 (**D**), total DRP1 (**E**), FIS1 (**F**), pDRP1^S616^ (**G**), and the pDRP1^S616^/DRP1 ratio (**H**) in microglia. Protein levels were normalized to β-actin, and the pDRP1^S616^/DRP1 ratio was calculated by normalizing pDRP1^S616^ to total DRP1. All values were expressed relative to the control group, which was set to 1 (n = 6). HBO treatment selectively prevented the reduction in MFN2 levels in L/N-stimulated microglia without significantly altering the levels of other detected mitochondrial fusion proteins. Moreover, HBO treatment inhibited DRP1 phosphorylation at Ser616 without affecting total DRP1 or FIS1 levels. One-way ANOVA followed by Tukey’s multiple comparisons test was used for (**B**–**D**,**F**), and the Kruskal–Wallis test followed by Dunn’s multiple comparisons test with Benjamini, Krieger, and Yekutieli FDR correction was used for (**E**,**G**,**H**). Data are presented as mean ± SD. * *p* < 0.05, ** *p* < 0.01. Con: control; L/N: lipopolysaccharide/nigericin; HBO: hyperbaric oxygen; MFN1: mitofusin 1; MFN2: mitofusin 2; OPA1: optic atrophy 1; DRP1: dynamin-related protein 1; FIS1: mitochondrial fission 1 protein.

L/N stimulation markedly downregulated MFN1, MFN2, and L-OPA1 expression in microglia. However, HBO treatment selectively prevented the reduction in MFN2 expression compared with L/N-stimulated microglia, while it did not significantly affect MFN1 or L-OPA1 levels, as shown by Western blot analysis (Figure 3A–D; *p* < 0.05 for MFN2). Regarding fission-related proteins, neither L/N stimulation nor HBO treatment significantly altered total DRP1 or FIS1 expression (Figure 3A,E,F). However, HBO treatment inhibited DRP1 phosphorylation at Ser616, a critical modification associated with mitochondrial fission [13], as reflected by both pDRP1^S616^ levels and the pDRP1^S616^/DRP1 ratio (Figure 3A,G,H; *p* < 0.01). These findings indicate that HBO selectively preserves MFN2 expression and suppresses fission-associated DRP1 phosphorylation at Ser616 in microglia, which may contribute to reduced mitochondrial fragmentation.

### 2.4. HBO Regulates MFN2 Expression and DRP1 Phosphorylation at Ser616 Through a ROS-Dependent Mechanism in LPS/Nigericin-Stimulated Microglia

**Figure 4:**
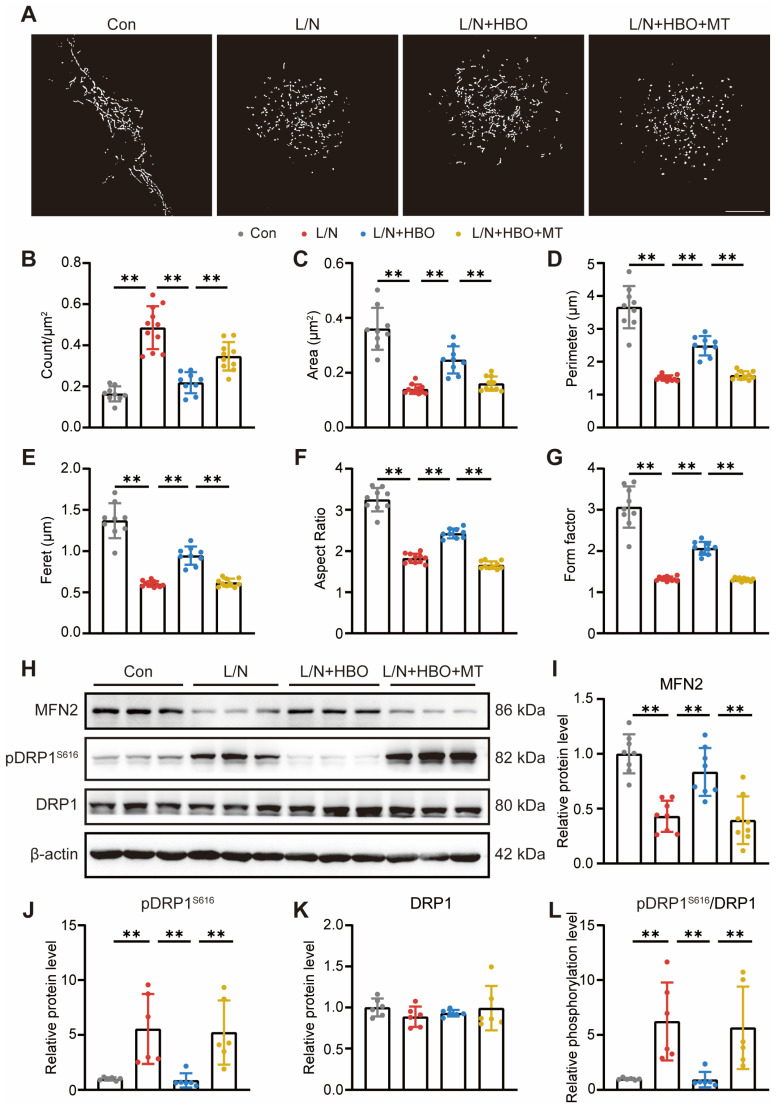
HBO regulates MFN2 expression and DRP1 phosphorylation at Ser616 through a ROS-dependent mechanism in LPS/nigericin-stimulated microglia: (**A**) Representative binarized TMRM-labeled images showing mitochondrial morphology in microglia treated with or without the mitochondrial ROS scavenger MitoTEMPOL (MT). Scale bar = 10 μm. (**B**–**G**) Quantitative analyses of mitochondrial parameters, including mitochondrial number per μm^2^ (**B**), mean area (**C**), mean perimeter (**D**), mean Feret diameter (**E**), aspect ratio (**F**), and form factor (**G**) (n = 9–11). Co-treatment with MT abolished the protective effect of HBO against L/N-induced mitochondrial fragmentation. (**H**–**L**) Representative immunoblot images (**H**) and quantitative analyses of MFN2 protein expression (**I**), DRP1 phosphorylation at Ser616 (pDRP1^S616^, **J**), total DRP1 expression (**K**), and the pDRP1^S616^/DRP1 ratio (**L**) in microglia. Protein levels were normalized to β-actin, and the pDRP1^S616^/DRP1 ratio was calculated by normalizing pDRP1^S616^ to total DRP1. All values were expressed relative to the control group, which was set to 1; n = 8 for MFN2 analysis; n = 6 for DRP1-related analyses. Co-treatment with MT also abolished the HBO-induced preservation of MFN2 protein levels and inhibition of DRP1 phosphorylation at Ser616. Welch’s ANOVA followed by Dunnett’s T3 multiple comparisons test was used for (**B**,**C**), one-way ANOVA followed by Tukey’s multiple comparisons test was used for (**D**–**G**,**I**), and the Kruskal–Wallis test followed by Dunn’s multiple comparisons test with Benjamini, Krieger, and Yekutieli FDR correction was used for (**J**–**L**). Data are presented as mean ± SD. ** *p* < 0.01. Con: control; L/N: lipopolysaccharide/nigericin; HBO: hyperbaric oxygen; MT: MitoTEMPOL; MFN2: mitofusin 2; DRP1: dynamin-related protein 1.

Our previous work showed that HBO mitigates neuroinflammation in a ROS-dependent manner [10]. To explore the role of ROS in HBO-mediated mitochondrial protection, we combined HBO treatment with the mitochondrial ROS scavenger MitoTEMPOL (MT). MT co-treatment reversed the inhibitory effect of HBO on L/N-induced mitochondrial fragmentation, as evidenced by increased mitochondrial number (Figure 4A,B; *p* < 0.01) and decreased mitochondrial area, perimeter, Feret diameter, form factor, and aspect ratio (Figure 4C–G; all *p* < 0.01), indicating impaired mitochondrial network integrity.

Moreover, MT co-treatment abolished the HBO-induced restoration of MFN2 expression in L/N-stimulated microglia (Figure 4H,I; *p* < 0.01). In addition, HBO treatment did not significantly affect total DRP1 protein expression but suppressed L/N-induced DRP1 phosphorylation at Ser616, and this effect was similarly reversed by MT co-treatment (Figure 4H,J–L; *p* < 0.01 for DRP1 phosphorylation at Ser616). These results indicate that mitochondrial ROS-related signaling is required, at least in part, for HBO-mediated preservation of MFN2 expression and suppression of DRP1 Ser616 phosphorylation, consistent with its role in suppressing microglial inflammatory activation in our previous report [10].

### 2.5. HBO Ameliorates Cerebral Ischemia–Reperfusion Injury and Regulates Mitochondrial Dynamics-Related Signaling Through a ROS-Dependent Mechanism In Vivo

**Figure 5:**
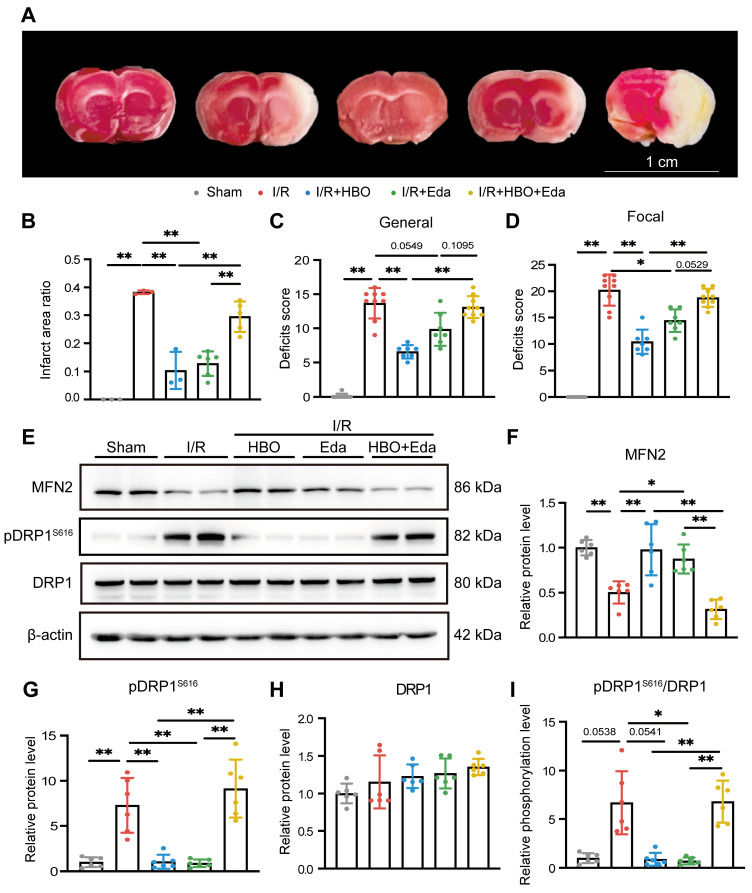
HBO ameliorates cerebral ischemia–reperfusion injury and restores the balance of mitochondrial dynamics through a ROS-dependent mechanism in vivo: (**A**) Representative TTC-stained brain sections from each group. Scale bar = 0.5 cm. (**B**) Quantification of infarct area in each group (n = 3–6). (**C**,**D**) General (**C**) and focal (**D**) neurological deficit scores of mice in each group (n = 7–9). Together, these results (**A**–**D**) suggest that the neuroprotective effect of HBO against cerebral ischemia–reperfusion injury is mediated, at least in part, by ROS-related signaling. (**E**–**I**) Representative immunoblot images (**E**) and quantitative analyses of MFN2 protein expression (**F**), DRP1 phosphorylation at Ser616 (pDRP1^S616^, (**G**)), total DRP1 levels (**H**), and the pDRP1^S616^/DRP1 ratio (**I**) in brain tissue. Protein levels were normalized to β-actin, and the pDRP1^S616^/DRP1 ratio was calculated by normalizing pDRP1^S616^ to total DRP1. All values were expressed relative to the control group, which was set to 1 (n = 6). The protective effects of HBO and Eda were associated with preserved MFN2 protein levels and reduced DRP1 phosphorylation at Ser616. Furthermore, the preservation of MFN2 expression and inhibition of DRP1 phosphorylation at Ser616 induced by HBO treatment were dependent, at least in part, on ROS-related signaling. One-way ANOVA followed by Tukey’s multiple comparisons test was used for (**B**), the Kruskal–Wallis test followed by Dunn’s multiple comparisons test with Benjamini, Krieger, and Yekutieli FDR correction was used for (**C**,**D**,**F**,**H**), and Welch’s ANOVA followed by Dunnett’s T3 multiple comparisons test was used for (**G**,**I**). Data are presented as mean ± SD. * *p* < 0.05, ** *p* < 0.01. I/R: ischemia/reperfusion; HBO: hyperbaric oxygen; Eda: edaravone; MFN2: mitofusin 2; DRP1: dynamin-related protein 1.

To further validate the ROS dependency of HBO-mediated protection in vivo, we used the tMCAO model and administered edaravone (Eda), a ROS scavenger. Both HBO and Eda significantly reduced infarct area after ischemia–reperfusion injury (Figure 5A,B; both *p* < 0.01 vs. I/R). In behavioral assessments, HBO significantly improved both the general neurological score and the focal deficit score compared with the I/R group (Figure 5C,D; both *p* < 0.01), whereas Eda improved the focal deficit score (*p* < 0.05) but did not significantly improve the general neurological score (*p* = 0.0549). HBO showed a numerically greater reduction in infarct area than Eda, although this difference was not statistically significant. Co-treatment with HBO and Eda significantly increased infarct area compared with either HBO or Eda alone (both *p* < 0.01). In neurological assessments, the combined treatment significantly worsened both general and focal scores compared with HBO alone (both *p* < 0.01), but did not significantly differ from Eda alone (*p* = 0.1095 for the general score and *p* = 0.0529 for the focal score). Moreover, the I/R + HBO + Eda group did not differ significantly from the I/R group, indicating that Eda largely abolished HBO-mediated protection. Together, these results indicate that HBO produced a more consistent protective profile across infarct and behavioral outcomes than Eda under the present experimental conditions, and suggest that ROS-related signaling is required, at least in part, for HBO-mediated protection after cerebral ischemia–reperfusion injury.

Consistent with our in vitro findings, Western blot analysis showed that both HBO and Eda prevented the I/R-induced reduction in MFN2 protein levels in the ipsilateral hemisphere (Figure 5E,F; both *p* < 0.05 vs. I/R). However, MFN2 levels were lower in the I/R + HBO + Eda group than in both the I/R + HBO and I/R + Eda group (Figure 5E,F; both *p* < 0.01), indicating that combined HBO and Eda treatment counteracted the MFN2-preserving effects observed with either treatment alone. Absolute pDRP1 Ser616 levels were increased after I/R and reduced by HBO or Eda treatment (Figure 5E,G; all *p* < 0.01 vs. I/R). In contrast, pDRP1^S616^ levels were higher in the I/R + HBO + Eda group than in either single-treatment group (Figure 5E,G; both *p* < 0.01). However, when normalized to total DRP1, the pDRP1^S616^/DRP1 ratio showed only a trend toward an increase after I/R compared with Sham mice (Figure 5I; *p* = 0.0538) and a trend toward a reduction after HBO treatment compared with the I/R group (Figure 5I; *p* = 0.0514). The pDRP1^S616^/DRP1 ratio was nevertheless higher in the I/R + HBO + Eda group than in either single-treatment group (Figure 5I; both *p* < 0.01). These results further support that HBO regulates mitochondrial dynamics-related signaling in vivo through a ROS-related mechanism, which may contribute to its neuroprotective effect.

## 3. Discussion

In the present study, we demonstrate that HBO attenuates microglial NLRP3 inflammasome activation and ameliorates cerebral ischemia–reperfusion injury, at least part through ROS-dependent regulation of mitochondrial dynamics. By systematically examining five key mitochondrial dynamics-related proteins, we identified MFN2 as a selective molecular target of HBO. MFN2 expression was specifically preserved by HBO treatment in LPS/nigericin-stimulated microglia, whereas MFN1, L-OPA1, total DRP1, and FIS1 remained largely unaffected. This selective preservation of MFN2, together with the concurrent suppression of DRP1 phosphorylation at Ser616, maintained mitochondrial network integrity and mitochondrial membrane potential, ultimately contributing to the inhibition of inflammasome activation. Importantly, these protective effects were attenuated by ROS scavenging with MitoTEMPOL in vitro and edaravone in vivo, supporting a role for ROS-related signaling as an important upstream mediator of HBO-induced protection. To our knowledge, this study provides the first evidence linking HBO-mediated neuroprotection to at least partly ROS-dependent preservation of MFN2 expression and suppression of DRP1 phosphorylation at Ser616 in microglia during cerebral ischemia–reperfusion injury.

Inhibiting mitochondrial fragmentation may represent a key mechanism underlying the protective effects of HBO therapy. A recent study demonstrated that HBO suppressed mitochondrial fission in endothelial cells subjected to oxygen–glucose deprivation injury [21], which is consistent with our findings in microglia. Notably, excessive mitochondrial fission is a hallmark of ischemia–reperfusion injury across multiple organs, including the brain [22], kidney [23], liver [24], and heart [25], and HBO has shown protective effects in these pathological contexts [26,27,28,29]. Beyond ischemia–reperfusion injury, excessive mitochondrial fission is also implicated in a broad spectrum of pathological states, including neurodegenerative diseases [30], metabolic disorders [31], cancer [32], liver injury [33], and high-altitude-related conditions such as cerebral edema and cognitive dysfunction [34,35]. In many of these conditions, inhibition of mitochondrial fragmentation has been shown to alleviate cellular or tissue injury, highlighting the importance of mitochondrial dynamics in maintaining cellular homeostasis. Consistently, HBO has demonstrated therapeutic potential in neurodegenerative diseases, including Alzheimer’s disease and Parkinson’s disease [36], metabolic disorders [37], cancer [38,39,40], liver injury [41], and high-altitude illnesses [42]. These observations raise the possibility that mitochondrial dynamics may represent a shared downstream target of HBO in multiple pathological settings, although this hypothesis requires disease-specific validation.

A key finding of this study is that HBO modulates both fusion- and fission-related markers of mitochondrial dynamics in microglia after cerebral ischemia–reperfusion injury. Specifically, HBO preserved MFN2 protein expression while suppressing DRP1 phosphorylation at Ser616, suggesting a dual regulatory mechanism that may rebalance mitochondrial fusion and fission. This reciprocal pattern is consistent with the observed reduction in mitochondrial fragmentation and mitochondrial dysfunction after HBO treatment, suggesting that HBO may restore mitochondrial homeostasis by rebalancing mitochondrial dynamics rather than by altering a single molecular event. Although direct evidence linking HBO to specific mitochondrial dynamics proteins remains limited, previous studies provide relevant support for this interpretation. In a heatstroke-induced brain injury rat model, HBO was shown to reduce DRP1 phosphorylation through the ROS/PKC pathway and improve thermoregulatory and neurological dysfunction [43]. In a rat model of myocardial ischemia–reperfusion injury, HBO pretreatment shifted the transcriptional profile of mitochondrial dynamics markers, increasing MFN1 and MFN2 mRNA levels while decreasing DRP1 mRNA levels, accompanied by reduced mitochondrial dysfunction and autophagy [44]. Building on these separate observations, our study demonstrates that HBO concurrently preserves MFN2 protein expression and suppresses DRP1 phosphorylation at Ser616 in microglia after cerebral ischemia–reperfusion injury, supporting coordinated mitochondrial dynamics remodeling as a mechanistic component of HBO-mediated neuroprotection.

ROS play a complex and context-dependent biphasic role in microglial activation. Excessive and sustained ROS overproduction can trigger inflammasome activation and exacerbate inflammatory responses, whereas moderate ROS elevation can act as an endogenous signaling mechanism that activates antioxidant defense systems and promotes mitochondrial adaptive remodeling [45,46]. The detrimental effects of excessive ROS provide a mechanistic rationale for the clinical use of edaravone in stroke treatment, consistent with our observation that edaravone alone alleviated ischemia–reperfusion injury and partially normalized mitochondrial dynamics markers in the present study. However, the protective effects of ROS modulation do not necessarily imply that ROS should be completely eliminated. Rather, HBO intervention has been reported to induce a transient and controllable increase in intracellular ROS levels [47], and our earlier work in a hyperglycemia-induced hemorrhagic transformation model showed that such moderate ROS elevation was indispensable for HBO-mediated neuroprotection [10]. Extending these findings to the present study, the attenuation of HBO-induced mitochondrial remodeling and neuroprotection by ROS scavengers, including MitoTEMPOL and edaravone, further suggests that adaptive ROS signaling is required for HBO-mediated mitochondrial dynamics remodeling and functional recovery. Together, these observations highlight the dual role of ROS as both a detrimental mediator and a beneficial signaling molecule, depending on its magnitude, duration, subcellular source, and regulatory context. This also suggests that combining HBO with potent antioxidants should be approached with caution, as excessive ROS scavenging may disrupt the adaptive redox signaling required for HBO-mediated mitochondrial protection and neuroprotection. Future studies are needed to define the specific ROS and subcellular sources that mediate HBO-induced protection, which may help optimize antioxidant strategies in patients receiving HBO therapy.

In considering the upstream oxygen-sensitive pathways that may couple HBO-induced ROS signaling to mitochondrial remodeling, hypoxia-inducible factor 1α (HIF-1α) emerges as a plausible candidate. Relevant to the present context, a recent study by Gong et al. linked HIF1A/HMOX1 signaling to microglial mitochondrial dysfunction and iron accumulation in a neuroinflammatory model, supporting a role for HIF-1α-related signaling in microglial mitochondrial health under stress-related conditions [48]. While the classical view links HIF-1α to hypoxic adaptation, emerging evidence indicates its broader involvement in oxidative stress responses and mitochondrial dynamics regulation within neural systems [49]. More directly, a HIF1α/PRDX1 axis was recently shown to promote DRP1-associated mitochondrial fragmentation through DRP1 deSUMOylation [50], a finding that connects HIF-1α-related signaling to DRP1-associated mitochondrial fission. Our previous work revealed that HBO treatment reduces HIF-1α expression in TGF-β-treated HFL1 cells [51], and similar regulation of HIF-1α signaling by HBO has been reported in other pathological settings [52]. Collectively, these findings support the hypothesis that HBO-mediated modulation of HIF-1α signaling may influence DRP1-associated mitochondrial fission in activated microglia, although its relationship with DRP1 Ser616 phosphorylation remains to be clarified.

In parallel with oxygen sensing, the KEAP1-NRF2 system represents a thiol-based redox sensor that coordinates antioxidant defense programs and maintains cellular redox homeostasis [53]. Notably, recent evidence links NRF2 pathway activation to the inhibition of microglial ferroptosis and the restoration of mitochondrial dynamic balance—including normalization of DRP1 and OPA1 expression—in cerebral ischemia–reperfusion injury [54]. This finding supports the concept that NRF2 signaling can preserve mitochondrial structural integrity and fission/fusion homeostasis in microglia under ischemic stress. Importantly, the NRF2–NLRP3 axis has been linked to mitochondrial health and innate immune regulation in neurodegenerative contexts [55], with broader implications for mitochondrial dynamics and mitophagy [56]. Mechanistically, ChIP-qPCR evidence suggests NRF2-dependent transcriptional modulation of Mfn2 [57], and functional studies link NRF2 to MFN2-related mitochondrial structural protection in brain injury [58]. Given that HBO has been reported to activate NRF2-related antioxidant signaling in spinal cord injury [59], and considering that the mitochondrial fission/fusion machinery is exquisitely sensitive to redox imbalance, it is plausible that HBO-induced NRF2 signaling contributes to MFN2 preservation and mitochondrial dynamics homeostasis, thereby helping to suppress NLRP3 inflammasome activation in activated microglia. However, whether HIF-1α or NRF2 directly mediates MFN2 preservation or DRP1 Ser616 dephosphorylation after HBO exposure in LPS/nigericin-stimulated microglia remains to be determined.

Despite these promising mechanistic findings, several limitations should be acknowledged. First, although MFN2 preservation and DRP1 Ser616 dephosphorylation were closely associated with reduced mitochondrial fragmentation and IL-1β release, their direct causal roles were not fully verified in this study. Future studies using MFN2 genetic manipulation and DRP1 loss- or gain-of-function approaches are needed to determine whether these mitochondrial dynamics regulators directly mediate the inhibitory effects of HBO on mitochondrial dysfunction and neuroinflammation. Second, although mitochondrial morphology and membrane potential were assessed in vitro using TMRM staining and morphological analysis, in vivo validation of mitochondrial structural and functional changes remains limited. Advanced imaging techniques, such as transmission electron microscopy, high-resolution confocal imaging, or in vivo mitochondrial imaging, would help clarify the translational relevance of HBO-mediated mitochondrial protection in ischemic stroke. Also, mitochondrial morphology was assessed using TMRM labeling, which depends on mitochondrial membrane potential. Although imaging and analysis conditions were standardized, future studies using membrane potential-independent mitochondrial markers or genetic mitochondrial reporters are needed to validate the morphological findings. Third, the upstream signaling pathways linking HBO-induced ROS to MFN2 preservation and DRP1 Ser616 dephosphorylation were not directly examined. Although HIF-1α and NRF2 are discussed as plausible oxygen- and redox-sensitive upstream signaling nodes, their direct regulatory relationships with MFN2 preservation and/or DRP1 Ser616 dephosphorylation remain unresolved. Future studies using pathway-specific pharmacological inhibitors, genetic knockdown or overexpression approaches, and temporal signaling analyses are needed to determine whether and how HIF-1α, NRF2, or other candidate pathways such as AMPK, ERK1/2, CDK1, and CaMKII participate in HBO-induced mitochondrial remodeling. Fourth, this study used only healthy young male mice, which does not fully reflect the demographic and pathological heterogeneity of clinical stroke patients. Future studies should include female and aged animals and clinically relevant comorbidity models, such as hypertension, diabetes, and hyperlipidemia, to better evaluate the generalizability and translational potential of HBO therapy.

## 4. Materials and Methods

### 4.1. Animals

Adult male ICR mice (8–12 weeks of age, 22–28 g) were obtained from the Experimental Animal Center of Nantong University (license number: SYXK (Su) 2024-0015). All animals were housed in individually ventilated cages under controlled environmental conditions (temperature 23–25 °C, relative humidity 55–75%, 12 h light/dark cycle) with free access to food and water. Mice underwent at least 3 days of habituation prior to experimentation to ensure physiological stability. All procedures were approved by the Institutional Animal Care and Use Committee of Nantong University (approval No. S20190920-303, 20 September 2019; and approval No. S20260226-009, 26 February 2026) and were conducted in accordance with the ARRIVE guidelines [60].

### 4.2. Transient Middle Cerebral Artery Occlusion (tMCAO) Model

The CIR model was established by transient middle cerebral artery occlusion (tMCAO). A total of 71 mice were used in this study. Before surgery, mice were randomly assigned to the following groups using a random number table. Because of the anticipated high postoperative mortality in the I/R and I/R + HBO + Eda groups, these groups were initially allocated larger numbers (I/R: n = 24; I/R + HBO + Eda: n = 11) to ensure sufficient surviving animals for analysis. The final sample size of each group was: sham group (n = 15), I/R group (n = 17), I/R + HBO group (n = 14), I/R + Eda group (n = 7), and I/R + HBO + Eda group (n = 9). The allocation of animals to specific endpoint assays (e.g., TTC, immunofluorescence, and Western blot) was determined before the surgery, and all surviving animals 24 h post-reperfusion underwent neurological scoring. The sample sizes for each specific assay are indicated in the figure legends. Due to the requirement for simultaneous HBO exposure, all animals in I/R + HBO and I/R + HBO + Eda were placed in the chamber together. Cage positions within the ventilated rack were randomised at the start of each batch to minimise positional bias. All outcome assessments were performed by an investigator blinded to group allocation.

Animals were allocated to different endpoint analyses, including neurological scoring, TTC staining, immunofluorescence, and Western blot, as indicated in the figure legends. For all surgical procedures, mice were anesthetized with isoflurane (Cat# R510-22-8, RWD, Shenzhen, China), with 5% for induction and 1.0–1.5% throughout surgery. The tMCAO model was induced as described previously [61]. Briefly, a midline cervical incision was made to expose the right common carotid artery (CCA), external carotid artery (ECA), and internal carotid artery (ICA). A silicone-coated monofilament (Cat# 602345PK5Re, Doccol, Sharon, MA, USA) was inserted via the ECA into the ICA until resistance indicated middle cerebral artery occlusion. The filament was secured, and the incision was sutured. After 1 h of occlusion, the filament was withdrawn to initiate reperfusion, followed by re-suturing of the incision. Sham-operated mice underwent identical surgical exposure except for filament insertion. Edaravone (Eda; 4.5 mg/kg, Changlong Yaoye, Tonghua, China), a ROS scavenger, was administered intraperitoneally 30 min before vessel occlusion. Body temperature was maintained at 37 °C throughout the procedure using a thermostatic heating pad. Mice were allowed to recover under warmed conditions before further treatment or sample collection. Model success was behaviorally confirmed immediately after reperfusion using the Bederson score [62]; only mice with scores of 2–3 and who survived 24 h post reperfusion were included in subsequent analyses.

### 4.3. Primary Microglia Culture

Primary microglia were prepared according to previously described protocols [10,61]. Neonatal ICR mice (P0–P3), obtained from the Experimental Animal Center of Nantong University, were used for microglial isolation. Cerebral cortices were dissected and digested with trypsin (Cat# 15090-046, Thermo Fisher Scientific, Waltham, MA, USA) at a final concentration of 0.05%, followed by centrifugation and resuspension. Cells were seeded into T75 culture flasks (Nest, Wuxi, China) and maintained for approximately one week, during which an astrocytic monolayer formed. The medium was subsequently replaced with growth medium supplemented with granulocyte–macrophage colony-stimulating factor (GM-CSF, 5 ng/mL, Cat# 78017, Stemcell Technologies, Vancouver, BC, Canada), and cultures were maintained for an additional week. Microglia were then harvested by shaking the flasks at 180 rpm for 30 min at 37 °C. The detached microglia were collected, centrifuged, counted, and plated onto poly-D-lysine-coated (PDL, Cat# P0899, Sigma-Aldrich, St. Louis, MO, USA) culture vessels. After 24 h of stabilization, the cells were used for subsequent experiments.

### 4.4. Microglial Inflammatory Model

Primary microglia were assigned to four experimental conditions: a control group without stimulation, a lipopolysaccharide/nigericin (L/N) group, an L/N + HBO group, and an L/N + HBO + MitoTEMPOL (MT) group. To activate the NLRP3 inflammasome, microglia in the L/N group were primed with lipopolysaccharide (LPS; 100 ng/mL, Cat# 00-4976-93, Invitrogen, Carlsbad, CA, USA) for 3 h, followed by stimulation with nigericin (10 μM, Cat# M7029, AbMole, Houston, TX, USA) for 1 h. In the L/N + HBO group, cells were exposed to HBO at 2.0 atmospheres absolute (ATA) for 1 h immediately after LPS priming and before nigericin stimulation. In the L/N + HBO + MT group, cells were pretreated with MT (20 μM, Cat# ab144644, Abcam, Cambridge, UK) before LPS priming, and MT was maintained throughout the subsequent LPS priming, HBO exposure, and nigericin stimulation steps. All microglial samples were harvested at designated endpoints for downstream analyses.

### 4.5. HBO Treatment

HBO treatment was carried out as previously reported [51]. Briefly, for animal experiments, HBO exposure was applied using a hyperbaric chamber designed for small animals. After flushing the chamber with pure oxygen for 5 min, the pressure was gradually increased to 2.0 ATA over 5 min, maintained for 1 h, and then slowly decompressed back to ambient pressure over another 5 min. The concentrations of carbon dioxide and oxygen were monitored by SDA carbon dioxide and oxygen monitors (Analox, North Yorkshire, UK) during the exposure, and the CO_2_ concentration was maintained below 2000 ppm by intermittent gas exchange. HBO treatment was applied immediately following filament withdrawal in the tMCAO model, whereas control animals remained in normobaric atmospheric air.

For in vitro studies, cultured microglia were placed in a hyperbaric chamber specifically designed for cell culture, with the internal temperature kept at 37 °C using a circulating water-based system. Cells were exposed to a 97.5% O_2_ and 2.5% CO_2_ gas mixture at 2.0 ATA, with CO_2_ partial pressure maintained at approximately 5 kPa to preserve physiological pH. HBO was administered for 1 h immediately following LPS stimulation. After HBO exposure, the cultures were returned to normoxic conditions until the designated collection time points.

### 4.6. 2,3,5-Triphenyltetrazolium Chloride (TTC) Staining

Mice were anesthetized with 2.5% Avertin (0.15 mL/10 g body weight; Aladdin, Shanghai, China) and euthanized by decapitation. Brains were rapidly removed, briefly frozen at −20 °C for 10 min, and coronally sectioned into five slices of 2 mm thickness using a pre-chilled brain matrix. To preserve the remaining ischemic brain tissues for Western blot analysis, TTC staining was performed on the second coronal slice; therefore, infarction was expressed as infarct area percentage rather than infarct volume. The second slice was incubated in 2% TTC (Cat# T8877, Sigma-Aldrich) solution in a 37 °C water bath for 5–10 min, rinsed gently with phosphate-buffered saline (PBS), and fixed in 4% paraformaldehyde (PFA) overnight. For imaging, slices were placed on a glass slide, excess liquid was removed, and photographs were taken against a white or black background. Infarct areas were quantified using Fiji software (Version 2.14.0/1.54f, NIH, Bethesda, MD, USA) by setting the scale and measurement parameters, applying a color threshold to distinguish viable from infarcted tissue, and calculating the infarct percentage as infarct area/total area of the selected slice × 100%.

### 4.7. Neurological Deficit Scoring

General and focal neurological deficits were assessed separately according to Clark’s scoring system [63] 24 h post-reperfusion. General deficits were scored based on hair appearance, ear position, eye condition, posture, spontaneous activity, and seizure-related behavior, whereas focal deficits were scored based on body symmetry, gait, climbing, circling behavior, forelimb symmetry, compulsory circling, and sensory response. Each score ranged from 0 to 28, with higher scores indicating more severe neurological impairment. Assessments were performed by investigators blinded to group allocation.

### 4.8. Immunofluorescence Staining

After fixation with 4% PFA and dehydration with 20% and 30% sucrose solution, brain tissues were cryosectioned into 25 μm coronal sections. Sections were rinsed in PBS and permeabilized with 0.3% Triton X-100 for 15 min at room temperature. Following permeabilization, sections were blocked with 10% bovine serum albumin for 2 h at room temperature and subsequently incubated overnight at 4 °C with the following primary antibodies: ASC (rabbit, 1:400, Cat# 67824, Cell Signaling Technology, Danvers, MA, USA, RRID: AB_2799736) and Iba1 (goat, 1:500, Cat# ab5076, Abcam, RRID: AB_2224402). After PBS rinsing, sections were incubated with appropriate fluorescent secondary antibodies for 2 h at room temperature in the dark. The secondary antibodies used were Alexa Fluor 488-AffiniPure Donkey Anti-Rabbit IgG (H + L) (donkey, 1:1000, Cat# 711-545-152, Jackson ImmunoResearch Labs, West Grove, PA, USA, RRID: AB_2313584), and Alexa Fluor 594-AffiniPure Donkey Anti-Goat IgG (H + L) (donkey, 1:1000, Cat# 705-585-147, Jackson ImmunoResearch Labs, RRID: AB_2340433). After secondary antibody staining, nuclei were counterstained with 4′,6-diamidino-2-phenylindole (DAPI) (Cat# C1006, Beyotime, Shanghai, China). Images were obtained with a Leica TCS SP8 confocal microscope (Wetzlar, Germany), and analyzed using Fiji software. The percentage of ASC-positive microglia was calculated as ASC^+^Iba1^+^ cells divided by total Iba1^+^ cells. Only the ASC signal localized in the cell body was counted when calculating the ASC^+^Iba1^+^/Iba1^+^ ratio.

### 4.9. Enzyme-Linked Immunosorbent Assay (ELISA)

The concentration of IL-1β released by primary microglia was quantified using a commercial ELISA kit (Cat# 70-EK201B/3-96, MultiSciences, Hangzhou, China) following the manufacturer’s instructions.

### 4.10. Mitochondrial Membrane Potential (MMP) Measurement and Mitochondrial Morphology Analysis

Image-iT™ TMRM (Tetramethylrhodamine Methyl Ester, 100 nM, Cat# I34361, Thermo Fisher Scientific) was added to the cell cultures during the final 30 min of treatment. After staining, cells were washed three times with PBS and then replaced with extracellular solution (Cat# C0216, Beyotime). Images were acquired using a Leica TCS SP8 confocal microscope.

For experiments aimed at MMP measurement, all imaging parameters (including laser power, detector gain, offset, and pinhole size) were strictly kept identical across all experimental groups to ensure quantitative comparability of TMRM fluorescence intensity. MMP was then evaluated by quantifying TMRM fluorescence intensity in individual cells using Fiji software.

For mitochondrial morphology analysis, imaging settings were optimized to visualize mitochondrial structures, and the same analysis pipeline was applied to all images within each experiment. Mitochondrial morphology was subsequently analyzed in Fiji using the Mitochondria Analyzer plugin [64,65,66,67].

The analysis workflow included conversion of images to 8-bit format, background subtraction, noise reduction, and enhancement of local contrast. Following thresholding to generate binary images, the following two-dimensional (2D) morphological parameters were analyzed: mitochondrial count, mean area, mean perimeter, and mean Feret diameter (long-axis length), which collectively describe mitochondrial size; form factor and aspect ratio were calculated to assess mitochondrial network complexity.

### 4.11. Western Blot

Western blotting was performed according to standard protocols. Briefly, protein samples were lysed using RIPA lysis buffer (Cat# P0013B, Beyotime) supplemented with 1% protease inhibitor mix (Cat# MB2678, Meilunbio, Dalian, China) and phosphatase inhibitor cocktail (Cat# 4906845001, Roche, Basel, Switzerland). Protein concentrations were determined using the Pierce BCA Protein Assay Kit (Cat# 23225, Thermo Fisher Scientific). For FIS1 detection, a 12% resolving gel was used; for all other proteins, a 10% resolving gel was used. A total of 20–40 μg of protein was loaded per lane. After electrophoresis, proteins were wet-transferred onto PVDF membranes. For FIS1 detection, 0.22 μm PVDF membranes (Cat# 3010040001, Roche, Basel, Switzerland) were used; for all other proteins, 0.45 μm PVDF membranes (Cat# IPVH00010, Sigma-Aldrich) were used. The membranes were blocked with 5% non-fat milk for 2 h at room temperature, followed by overnight incubation at 4 °C with primary antibodies.

The primary antibodies used were as follows: MFN2 (rabbit, 1:1000, Cat# BM4906, Boster, Wuhan, China, RRID: AB_3741795), MFN1 (rabbit, 1:1000, Cat# ab221661, Abcam, RRID: AB_2941083), OPA1 (rabbit, 1:1000, Cat# 80471, Cell Signaling Technology, RRID: AB_2734117), DRP1 (rabbit, 1:1000, Cat# 8570, Cell Signaling Technology, RRID: AB_10950498), phospho-DRP1 (Ser616) (rabbit, 1:1000, Cat# M00556-4, Boster, RRID: AB_3751370), FIS1 (rabbit, 1:1000, Cat# A01932-2, Boster, RRID: AB_3081504), and β-actin (mouse, 1:10,000, Cat# A5316, Sigma-Aldrich, RRID: AB_476743). After washing, the membranes were incubated with HRP-conjugated secondary antibodies for 2 h at room temperature. The following HRP-conjugated secondary antibodies were used: goat anti-mouse IgG (1:10,000 dilution; Cat# BL001A, Biosharp, Beijing, China, RRID: AB_2827665) and goat anti-rabbit IgG (1:10,000 dilution; Cat# BL003A, Biosharp, RRID: AB_2827666).

Protein bands were visualized using an ECL detection system (Cat# E412-02, Vazyme, Nanjing, China) and captured using a Tanon 5200 Multi-Imaging System (Shanghai, China). Band intensities were quantified using Fiji software. Western blot band intensities were quantified using integrated intensity, defined as the total signal intensity within the selected band region, and normalized to the corresponding loading control. Total protein levels and pDRP1^S616^ band intensity were first normalized to β-actin. The pDRP1^S616^/DRP1 ratio was then calculated to evaluate DRP1 phosphorylation relative to total DRP1. Relative protein levels were expressed as fold changes relative to the mean value of the control group, which was set to 1.

### 4.12. Statistical Analysis

Sample size was calculated using statistical power analysis in accordance with the 3R principles for laboratory animals. The primary endpoints for sample size estimation were the differences in general and focal neurological deficit scores between the I/R and I/R + HBO groups. Effect sizes were derived from preliminary data using an online calculator [68] (https://www.campbellcollaboration.org/calculator, accessed on 14 February 2026), based on means, standard deviations, and sample sizes, with both Cohen’s d and Hedges’ g exceeding 2.0, suggesting a large effect size. Sample size was then computed in G*Power software [69] (version 3.1.9.7) for one-way ANOVA based on the primary three-group comparison, with effect size f = 2.0, α = 0.05, power = 0.95. The calculation yielded a minimum sample size of 3 animals per group. Considering potential surgical mortality, model failure, and variability in outcome measures, larger group sizes were used where appropriate, as indicated in the figure legends.

Data were analyzed using GraphPad Prism (v10.1.1, GraphPad Software, Boston, MA, USA). Normality was evaluated using the Shapiro–Wilk test, and equality of variances was examined by the Brown–Forsythe test. Data are presented as mean ± standard deviation (SD). For comparisons involving three or more groups, parametric methods were applied when the normality and homogeneity of variance assumptions were satisfied, using one-way ANOVA followed by Tukey’s test for pairwise comparisons. In cases of unequal variances, Welch’s ANOVA with Dunnett’s T3 post hoc test was employed. When normality was violated, the nonparametric Kruskal–Wallis test was used. Post hoc pairwise comparisons were conducted using Dunn’s test, with *p* values adjusted for false discovery rate using the two-stage step-up method of Benjamini, Krieger, and Yekutieli. For each experimental panel, the specific statistical test applied is indicated in the corresponding figure legend. Reported *p* values were adjusted for multiple comparisons where applicable. Two-tailed *p* values < 0.05 were considered statistically significant.

## 5. Conclusions

In conclusion, this study demonstrates that HBO attenuates microglia-mediated neuroinflammation after ischemic stroke by suppressing NLRP3 inflammasome activation through adaptive ROS-dependent regulation of mitochondrial dynamics. Mechanistically, HBO preserves MFN2 expression and suppresses DRP1 Ser616 phosphorylation, thereby maintaining mitochondrial integrity and membrane potential. These findings advance our understanding of HBO-mediated neuroprotection and support the development of HBO as a non-invasive therapeutic strategy for ischemic stroke, while also providing translational guidance that concomitant use with potent antioxidants should be approached with caution to avoid interfering with adaptive ROS signaling of HBO.

## Data Availability

The original contributions presented in this study are included in the article. Further inquiries can be directed to the corresponding author.

## Abbreviations

The following abbreviations are used in this manuscript:

ASC	Apoptosis-associated speck-like protein containing a CARD
CCA	Common carotid artery
CIR	Cerebral ischemia–reperfusion
DAPI	4′,6-diamidino-2-phenylindole
DRP1	Dynamin-related protein 1
ECA	External carotid artery
Eda	Edaravone
ELISA	Enzyme-linked immunosorbent assay
FIS1	Mitochondrial fission 1
GM-CSF	Granulocyte-macrophage colony-stimulating factor
HBO	Hyperbaric oxygen
Iba1	Ionized calcium-binding adapter molecule 1
ICA	Internal carotid artery
IL-1β	Interleukin-1 beta
I/R	Ischemia/reperfusion
LPS	Lipopolysaccharide
L/N	Lipopolysaccharide and nigericin
L-OPA1	Long isoform of OPA1
MFN1	Mitofusin 1
MFN2	Mitofusin 2
MMP	Mitochondrial membrane potential
MT	Mito-TEMPOL
NAC	N-acetyl-L-cysteine
NLRP3	NOD-like receptor protein 3
OPA1	Optic atrophy 1
PDL	Poly-D-lysine-coated
PFA	Paraformaldehyde
ROS	Reactive oxygen species
tMCAO	Transient middle cerebral artery occlusion
TMRM	Tetramethylrhodamine methyl ester
TTC	2,3,5-triphenyltetrazolium chloride
